# Effect of Intelligent Operating Hole Towel in Cataract Patients

**DOI:** 10.1155/2022/2638428

**Published:** 2022-08-18

**Authors:** Suhui Xu, Meijuan Lan, Haiyan Cai, Pei Zhang

**Affiliations:** Second Affiliated Hospital Zhejiang University College of Medicine, Ophthalmology Department, No. 88 Jiefang Road, Shangcheng District, Hangzhou, Zhejiang 310007, China

## Abstract

**Backgrounds:**

The eyeball and its ancillary tissues are important organs with the same shape and structure, and examining the surgical site is particularly important in ophthalmic surgery. A safe and easy-to-operate ophthalmic surgical hole towel is of great significance for improving the safety of ophthalmic surgery.

**Objective:**

To explore the effect of intelligent operating hole towel in cataract patients.

**Methods:**

From April 2020 to April 2021, 1220 cases of cataract patients who needed surgery in the second affiliated hospital Zhejiang University college of medicine, were recruited and randomly divided into the control group and the observation group. The control group adopted a disposable ophthalmic single-port operation cloth, and the intelligent surgical hole towel was used in the observation group. Incidences of surgical site errors, the amount of operation time, bacterial infections, and patient satisfaction were recorded.

**Results:**

The average operation time in the observation group had obviously reduced compared with the control group (*p* < 0.05). Moreover, patients' overall medical satisfaction in the observation group improved significantly compared with the control group (*p* < 0.05).

**Conclusions:**

The design and use of the new intelligent ophthalmic surgical hole towel can promote the efficiency of ophthalmic surgery, realize the intelligent verification of surgical eye, reduce the risk of surgical site errors and improve medical safety.

## 1. Introduction

Under the theme “Safe Surgery, Save Lives,” the 2007–2008 WHO Alliance for patient safety aims to improve surgical safety and save more lives worldwide. The Surgical Safety Checklist emphasizes that Surgical Safety checks include “before anesthesia (Sign in),” “before surgery (Time out),” and “before patients leave the operating room (Sign out).” WHO recommends the continuous optimization and development of surgical safety verification systems based on specific surgical procedures to ensure that the correct surgical site is marked on the patient's body and that the surgery is performed on the correct body part [1].

A cataract is an eye disease with clouding of the lens. With the aging of the global population, the incidence of cataracts is rising as the top treatable blinding eye disease worldwide. Since the rapid pace of eye surgery and the special anatomy of the eyeball, it is easy for the surgeon to confuse the eyes when covering the patients' eyes after the surgical field is disinfected, leading to the wrong covering of the eyes and causing negative effects. In case of surgical field placement error, the surgical staff can timely find the error of surgical field placement during the “Time Out” safety check and suspend the operation. Although the surgical site error did not occur due to the timely intervention, ophthalmic surgery is mainly performed under local anesthesia. Patients are conscious that the error of eye tissue placement can easily cause a trust crisis of patients. Moreover, redisinfecting towels and preparing all kinds of products increased the surgical staff's workload and affected the operation's efficiency [2]. In addition, the surgical towel is incorrect, and the surgical safety staff is not making proper checks leading to the surgical site error unidentified before “Time Out,” which may result in serious adverse events occurring. Therefore, “check the eyes when placing surgical towels” is significantly needed.

Before ophthalmology patients enter the operating room, medical staff confirm the patient's identity by using the patient's name and medical record number. The surgeon checks the medical record and image data to verify the surgical safety of the surgical eyes [3]. In the procedure of surgical safety check, a new type of intelligent ophthalmic surgical hole napkin is added to assist in the surgical eye identification check. It can not only improve the visual perception of the surgical eye but also prevent the identification errors of the surgical site in the process of surgery [4, 5]. The new intelligent ophthalmic operating towel is based on the disposable ophthalmic operating towel and adds intelligent design in the key link of “operation cloth.” Currently, the surgical marker used in ophthalmology is an oily marker, which can only be used for cosmetic surgical markers [6, 7]. Special intelligent material is used in the new special intelligent surgical marker pen, which can react with the intelligent material of the surgical hole towel when completing the marking [8, 9]. The voice chip is embedded in the new smart surgical towel. When the surgical towel is unfolded, the voice prompts, “Please check the surgical eye.”

The disadvantage of the previous ophthalmic surgical towel is that it cannot be directly positioned on the front of the surgical site and usually requires another person to locate and operate correctly, which has the disadvantages of complex operation, easy contamination, and dispersion of surgical equipment preparation. Moving the towel hole caused a lot of inconvenience to the operation, extended the operation time, and increased the number of operation steps. It is necessary to design a towel to achieve the purpose of safety, convenience, and convenient operation. This study intends to promote the efficiency of ophthalmic surgery, realize the intelligent verification of surgical eye, reduce the risk of surgical site errors and improve medical safety through the design and use of the new intelligent ophthalmic surgical hole towel.

## 2. Methods

### 2.1. Participants

From April 2020 to April 2021, 1220 cases of cataract patients in the second affiliated hospital Zhejiang University were selected and randomly divided into two groups. There were 248 males and 362 females in the control group and 266 males and 344 females in the observation group. All patients in this study completed this study and no patients who dropped out halfway.

### 2.2. Inclusion and Exclusion Criteria

Inclusion criteria: (1) patients with cataracts need surgery; (2) no other serious diseases such as heart, liver, kidney, and other important organ dysfunction; (3) patients voluntarily participated and signed informed consent forms. Exclusion criteria: (1) patients with cataracts do not need surgery; (2) complicated with serious diseases such as heart, liver, kidney, and other important organ dysfunction; (3) poor treatment compliance; (4) incomplete clinical data or withdrawal; (5) not signing informed consent.

### 2.3. Methods

#### 2.3.1. The Control Group

Disposable ophthalmic single-port operation hole towel was adopted in the control group. Before the operation, the skin of the eye is disinfected and the patient's hair is covered with a headband. The disposable ophthalmic operation cloth includes the main body of the cloth and a single oval surgical eye hole. The back of the main body of the cloth is located on both sides of the surgical hole with glue, which is glued and fixed simultaneously. The surgical opening directly faces the patient's surgical eye, and the surgical towel is unfolded in turn according to the arrow pointing at the front of the towel.

#### 2.3.2. The Observation Group

Intelligent surgical hole towels were used in the observation group. After the skin around the eyes is disinfected, the patient's hair is covered with a turban. Then, the procedure of towel laying for surgical holes follows the control group. When the operating hole towel was expanded to contact the operating eye with the intelligent operating mark, the voice suggested that the operating site was correctly exposed.

### 2.4. Observe Indicators

The data of surgical site errors, amount of the operation time, bacterial detection, and patient satisfaction were recorded.

### 2.5. Statistical Analysis

The SPSS 19.0 statistical software was employed to analyze data. The count data was expressed by the number of cases and percentage (%), and the *χ*^2^ test performed the comparison between groups, *t*-test was used in independent samples. *p* < 0.05 indicates that the difference was statistically significant.

## 3. Results

### 3.1. Schematic Diagram of Surgical Towel and Marker Pen

**Figure 1:**
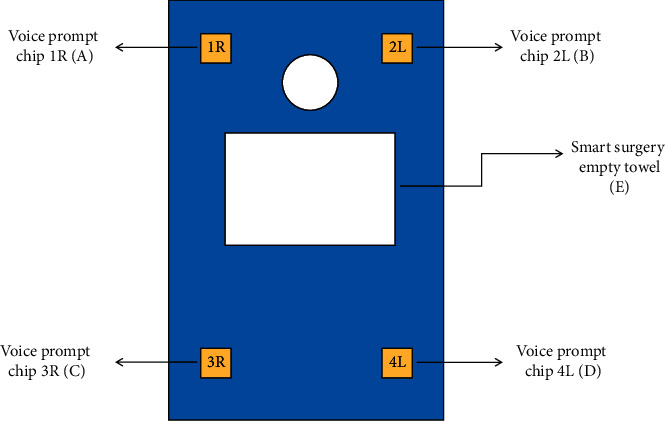
New intelligent ophthalmic surgical hole towel.

As shown in Figure 1, the new intelligent ophthalmic surgical hole towel is displayed. Figure 1 A, B, C, and D represent voice prompt chips, and E refers to the smart surgery empty towel hole.

### 3.2. Basic Characteristics of the Patients

One thousand two hundred twenty cases of cataract patients who needed surgery were selected from April 2020 to April 2021 in the second affiliated hospital Zhejiang university hospital. The basic characteristics of the patients, including age, gender, and education level showed no statistical difference (Table 1).

### 3.3. The Incidents of Surgical Site Errors after Towel Placement and Surgical Site Errors

5 cases of surgical site errors after towel placement were found in the control group, and no surgical site errors after towel placement were found in the observation group. Although compared between the two groups (the distinction does not prove a statistical significance, *p* > 0.05), it can be seen that the number of surgical site errors after towel placement found in the observation group was significantly lower than in the control group. Notably, there are no surgical site errors found in the observation group and the control group (*p* > 0.05) (Table 2).

### 3.4. Comparison of the Amount of Operation Time between the Two Groups

The average amount of operation time in the observation group is 20.93 ± 3.556 (min) and the average amount of operation time in the control group is 21.98 ± 4.127 (min). Results indicated the average amount of the operation time in the observation group had obviously contracted, *p* < 0.05, with a statistical difference (Table 3).

### 3.5. The Comparison of Bacterial Detection between the Two Groups

There was no statistically significant difference in bacterial detection between the two groups (*p* > 0.05). Results showed that only two bacterial infections occurred in the control group and bacterial infections were detected in the observation group (Table 4).

### 3.6. Comparison of the Patients' Satisfaction between the Two Groups

The number of patient satisfaction in the observation group is 600 and the number of patient satisfaction in the control group is 585. Results suggested that patients' overall medical satisfaction in the observation group was improved by applying a new intelligent ophthalmic surgical hole towel combined with a surgical site marker pen (Table 5). The difference was a statistically significant comparison of the patient satisfaction between the two groups (*p* < 0.05).

## 4. Discussion

Medical and health safety is a major issue affecting the health of hundreds of millions of people. With the improvement of people's economic level, more and more attention was focused on health. Meanwhile, higher requirements on the level and quality of medical and health services were put forward to the hospital. The eyes are the main organ for humans to collect information from the outside world. More than 80% of information is obtained from the eyes, so the average person attaches much more importance to their own eyes than other organs. Ophthalmology, as a special specialty, is characterized by the fact that most ophthalmic diseases need surgical treatment. Only a few can be treated with simple drugs or physical therapy. Therefore, the ophthalmic medical service is based on surgical treatment, supplemented by drugs and physical therapy. Surgery is needed to treat ocular plastic surgery, infection, trauma, tumor, cataract, and optometry. Surgical treatment not only involves equipment, instruments, and consumables but also involves the participation of medical and anesthesia professionals. And, due to the small surgery scale, the number of operations is far more than in other surgical specialties. In this way, the management of ophthalmic surgery is more strict and the quality of medical treatment is higher.

A cataract is the world's number one blinding eye disease. Cataracts are mainly because by lens metabolism disorder, lens protein denaturation, formation of turbidity, and blindness. With the rapid aging of the global population, the incidence of cataracts is increasing. According to incomplete statistics from the World Health Organization, there are about 500,000 new cataract blind people yearly [10, 11]. Currently, the number of cataract blindness in China is second only to India, ranking second in the world. With the development of health care, cataract surgery can restore nearly normal vision in most patients [12, 13]. Improving patients' visual ability and recovery is the key to improving patients' quality of life.

Surgical towels are the basic operation before the operation begins. Surgical pads not only keep the surgical area sterile but also prevent skin bacteria from entering the incision, thereby reducing the risk of surgical site infection. The disadvantage of the original ophthalmic surgical towel is that it is not conducive to the safety verification of the surgical site. In the process of replacing the surgical towel, it will not only cause a lot of inconvenience to the surgical operation but also prolong the operation time, increase the number of operation steps, and significantly increase the possibility of contamination of the surgical area, which is easy to cause intraoperative infection [14]. In addition, ophthalmic surgery anesthesia is mainly performed under local anesthesia. Patients are conscious during the operation and are more prone to psychological stress reactions such as tension and anxiety under closed and dim operation orders for a long time. Therefore, it is necessary to improve. In this study, a kind of ophthalmic operation cloth is proposed. Although its structure is complex, it can realize intelligent operation site verification and is convenient for operation preparation and operation.

In 1957, the United States medical and health institutions on the health service patient satisfaction evaluation for the first time. Measuring patient satisfaction is the establishment and constitutes a prerequisite for hospital certification. Patient satisfaction has become a deterministic index of the quality and effect of the health service system. Patient satisfaction with surgery is considered an important factor affecting patients' overall medical satisfaction with the hospital. In the present study, the use of the new intelligent operating hole towel can promote the efficiency of ophthalmic surgery, realize the intelligent verification of surgical eye, reduce the risk of surgical site errors and improve medical safety. In addition, results showed that patients' overall medical satisfaction in the observation group was obviously improved compared with the control group. When high-quality medical services are advocated, high-quality medical technology can make patients feel safe and increase their trust in doctors.

There are also some shortcomings in this study. All the physicians were from the same surgical team in the second affiliated hospital Zhejiang university. Therefore, the results of this survey cannot fully represent the overall level and status of ophthalmic surgery in China. It is still necessary to expand the study's sample size and conduct followup statistics.

## 5. Conclusions

The intelligent operating hole towel gives the surgeon a more intuitive experience so that it can be attached to the patient's surgical site more quickly and accurately. The practicability of an intelligent operating hole towel can not only shorten the operation time to reduce the risk of surgical site infection but also strengthen the aseptic barrier to ensure the safety of patients, which is worthy of clinical promotion.

## Figures and Tables

**Table 1:** Comparison of basic characteristics of the patients between the two groups.

Basic indexes	Observation group (*n* = 610)	Control group (*n* = 610)	*X* ^2^	*p*
Age (year)				
65–70	115	126	1.835	0.402
60–65	358	365		
55–60	137	119		

Sex				
Male	266	248	1.089	0.324
Female	344	362		

Education level				
≤Primary school	371	394	1.873	0.392
Middle-high school	159	145		
>High school	80	71		

**Table 2:** The incidences of surgical site errors were compared between the two groups.

Groups	Observation group	Control group	*X* ^*2*^	*p*
Surgical site errors after towel placement	0	5	5.021	0.062
Surgical site errors	0	0	0	1
*n*	610	610	—	—

**Table 3:** Comparison of amount of the operation time between the two groups.

Groups	Amount of the operation time	*t*	*p*
Observation group	20.93 ± 3.556	4.749	*P* < 0.001
Control group	21.98 ± 4.127		

**Table 4:** Comparison of bacterial detection between the two groups.

Groups	Bacterial detection			*X* ^*2*^	*p*
	*N*	Cases	Incidence (%)		
Observation group	610	0	0	3.007	0.249
Control group	610	2	0.328		

**Table 5:** Comparison of the patients satisfaction between the two groups.

Groups	Patients satisfaction			*X* ^2^	*p*
	*N*	Cases	Incidence (%)		
Observation group	610	600	98.361	6.618	0.015
Control group	610	585	95.902		

## Data Availability

The datasets used and analyzed during the current study are available from the corresponding author upon reasonable request.
